# A facile synthesis of porous graphene for efficient water and wastewater treatment

**DOI:** 10.1038/s41598-018-19978-8

**Published:** 2018-01-29

**Authors:** Tanveer A. Tabish, Fayyaz A. Memon, Diego E. Gomez, David W. Horsell, Shaowei Zhang

**Affiliations:** College of Engineering, Mathematics and Physical Sciences, University of Exeter, Exeter, EX4 4QF United Kingdom

## Abstract

The use of two-dimensional graphene-based materials in water treatment has recently gained significant attention due to their unique electronic and thermal mobility, high surface area, high mechanical strength, excellent corrosion resistance and tunable surface chemistry. However, the relatively expensive, poor hydrophobicity, low adsorption capacity and recyclability, and complex post-treatment of the most pristine graphene frameworks limit their practical application. Here, we report a facile scalable method to produce highly porous graphene from reduced graphene oxide via thermal treatment without addition of any catalyst or use of any template. Comparing to conventional graphene counterparts, as-prepared porous graphene nanosheets showed evident improvement in hydrophobicity, adsorption capacity, and recyclability, making them ideal candidate materials for water treatment. Superhydrophobic and superoleophilic porous graphene prepared in this work has been demonstrated as effective absorbents for a broad range of ions, oils and organic solvents, exhibiting high selectivity, good recyclability, and excellent absorption capacities > 90%. The synthesis method of porous graphene reported in this paper is easy to implement, low cost and scalable. These attributes could contribute towards efficient and cost-effective water purification and pollution reduction.

## Introduction

Heavy metals, organic solvents, toxic organic dyes, organic chlorine compounds and solvent oils are primary pollutants of water resources^1^. For example, the total amount of oil lost to the environment through tanker incidents from 2010 to 2016 was 39,000 tonnes^2^. Additionally, oil and grease are major pollution sources of road runoff. Excessive concentration of Arsenic, Fluoride, Nitrate, Selenium and Chromium in groundwater are a global concern, making groundwater resources unfit for human consumption and resulting in several health complications^3,4^. Also, over 15% of the world’s total production of dyes is released as waste during the dyeing of fabrics^5^. These pollutants are not readily biodegradable and their accumulation in living organisms has profound biological implications. Due to the toxic effects of these contaminants in geochemical systems, a variety of techniques have been utilized to eliminate them, including flocculation^6^, membrane filtration^7^, solvent extraction^8^, biosorption^9^, chemical precipitation^10^, reverse osmosis^11^, and adsorption^12^. Among these techniques, the adsorption technique is considered to be highly efficient and economical. Moreover, adsorption method is the best suited for studying the accumulation of ions, oils and organic dyes in porous networks. Various materials such as carbon nanotubes, activated carbon, clays^13–15^, plant wastes^16^, and agricultural byproducts^17,18^ have been extensively explored as adsorbents to remove the pollutants mentioned above from water, but they all suffer from poor adsorption selectivity, low yield, slow adsorption kinetics, and poor recyclability and regeneration. Also, their fabrication processes are expensive, complex, and difficult to scale up for practical use^19^. On the other hand, a great deal of attention has recently been attracted to explore solutions for the problems related to severe water pollution arising from natural pollution processes of geochemical systems^20^. Therefore, it is necessary to explore alternative novel materials which can overcome the disadvantages of the conventional adsorbents stated above.

Graphene, a single atom thick sheet of sp^2^ hybridized carbon atoms arranged in a two-dimensional honeycomb lattice structure is an ideal adsorbent with the highest known surface area^21,22^. Recent experimental investigations have shown that graphene-based materials have a high adsorption capacity^23^. Nevertheless, recent developments in the preparation of porous graphene (PG) offer new opportunities to design advanced materials with enhanced adsorption capacities and reusability owing to their microporous and mesoporous interconnected network which allows access and diffusion of ions and molecules^24–26^. Conventional ways to prepare PG include electron beam irradiation, ion bombardment, doping, templating, chemical etching, chemical vapour deposition and other chemical methods^27–29^. Unfortunately, these techniques are very expensive and not viable for practical applications as with these techniques, small and non-uniform amounts of porous structured graphene are created via complex routes such as physical ion beam irradiation and organic synthesis. Therefore, it still remains a major challenge to uniformly introduce pores in a graphene sheet. PG can be produced on a large scale by conversion of chemically synthesized graphene oxide (GO) into porous reduced graphene oxide (rGO) via reduction and thermal treatment. Theoretically, graphene has a specific surface area (SSA) of 2630 m^2^ g^−1^^24^. However, experimentally determined values for porous rGO are generally less than 100 m^2^ g^−1^^24–26^. Zhang *et al*. have demonstrated the use of potassium hydroxide to activate rGO and make it porous to increase the SSA, interlayer spacing of graphite, adsorption capacity and supercapacitance^30^. Santosh *et al*. reported N-doped porous rGO for supercapacitors with an SSA of 206.62 m^2^g^−1^ ^31^. While the conventional methods are effective, they introduce foreign chemicals that are difficult to remove. Unfortunately, the final products obtained from few studies were essentially oxidized porous graphite sheets rather than the claimed porous reduced graphene oxide nanosheets^25–28^. It is therefore necessary to design the novel synthesis approaches to the scalable preparation of porous graphene to use in a variety of applications. Highly porous nanosheets have the potential to overcome all of these issues. PG comprises few-layered planar graphene sheets with a unique combination of high surface area, super hydrophobicity, uniform porous architecture, optical transparency, high oxidation resistance and chemical stability^32,33^. Such planar porous nanostructures provide an easy access for ions, facilitating faster adsorption and desorption of contaminants, and subsequently improve both adsorption rate and adsorption capacity, hence making them highly effective adsorbents of metal ions^34^ as well as dissolved organic pollutants^35^. PG could provide a significant robustness in the porous assemblies to link the defects for contaminant uptake. Furthermore, it could have a high structural stability which is essential during the adsorption and desorption process so that it can be collected easily and used repeatedly.

In this study, we present a facile cost-effective production route for this novel superhydrophobic PG based on thermal treatment of rGO, so as to unlock the current main bottlenecks in its commercial application. Porous nanosheets with a surface area of 652 m^2^/g was achieved by introducing and disintegrating a small number of edge defects into the PG by thermal treatment of rGO. Additionally, the temperature range involved in this treatment process was (190–200 °C), which was lower than that previously reported for the synthesis of porous rGO (800 °C^30^). Chemically synthesized GO was reduced using hydrazine to rGO, which was then thermally treated to produce PG. Surface area of as-prepared PG was among the highest reported for porous rGO to date (652 m^2^ g^−1^). Moreover, graphene’s physiochemical, mechanical, electrical and thermal characteristics including high conductivity, high surface area, porous morphology and chemical inertness make them not only ideal candidates for water treatment but also potentially viable alternatives for other applications such as for batteries, photocatalysis, DNA sequencing, and enzyme modulation. As-prepared PG provided abundant ion/porous channels, showing an excellent selective adsorption capacities and fast adsorption kinetics for the elimination of arsenic, fluoride, nitrate, oil, methylene blue and rhodamine B. Systematic studies on the adsorption performances of PG were carried out, using different thermodynamic and kinetic models, and Fourier transform infrared (FTIR), X-ray diffraction (X-Ray) and elemental mapping analysis were carried to in order to elucidate the adsorption mechanisms. PG was revealed to be highly effective for water decontamination, with superior adsorption capacity, kinetics, recovery, regeneration and recyclability.

## Materials and Methods

### Synthesis of porous graphene

Exfoliated graphite oxide flakes, with ~0.5–20 μm lateral size and ~1.5 nm thickness, were prepared following the modified Hummers method previously reported by us^36^. Graphite was initially oxidized to form graphite oxide, which was further exfoliated and chemically reduced to graphene sheets. The thickness of GO sheets varied between 0.8–1 nm. Before the reduction, 500 ml aqueous solution of graphite oxide with concentration of 1 mg/ml was synthesized, and further exfoliated for 2 h in an ultrasonic bath (Bandelin Sonorex RK-100H). The pH values were found to be in the range 9–11. 150 ml GO (1 mg/ml) was combined with 1.5 ml of hydrazine (35 wt%) under magnetic stirring in a flask which was heated to 100 °C. After 12 h reaction, hydrazine (1.5 ml) was again added to perform the reaction for additional 2 h to ensure full reduction of the GO. The rGO was allowed to settle, washed with distilled water and filtered until the supernatant became clear. To obtain porous nanosheets, the filtered product was oven-dried in vacuum overnight and then thermally treated at 200 °C in Ar for 12 h under a slow ramp rate of 3 °C min^−1^. The preparation scheme is schematically shown in Fig. S1 of supporting information (SI).

### Basic Characterization

Microstructures of PG, graphite flakes and GO samples were taken on a Philips XL-30 scanning electronic microscope (SEM) under high vacuum conditions with accelerating voltage 20 kV and the samples were mounted onto carbon sticky tape. High resolution microstructural images were also taken, along with elemental mapping analysis, on a JEOL-2100 transmission electron microscope (TEM) operating at an accelerating voltage 200 kV. The powder sample was dispersed in acetone, after which the sample was dropped on the centre of a carbon Cu grid using a micro pipet. The X-ray photoelectron spectrometer (XPS) spectra were recorded on an ESCALab 250 XPS using monochromated Al Kalph X-ray as the excitation source, and the Raman spectra were collected using a 532 nm laser excitation operating at 6 mW power. The power of the laser was kept at 6 mW. X-ray diffraction (XRD) analysis was performed using Cu Kα radiation (at 40 kV and –40 mA). Fourier-transform infrared (FTIR) spectra were obtained in the wavenumber range of 2000–500 cm^−1^ using a Bruker Optics Tensor-27 FTIR spectrometer. UV–Vis absorbance were obtained by using a Jenway 6715 UV/Vis spectrophotometer. Nitrogen gas sorption analysis was conducted using a Quantachrome Autosorb-iQ gas sorptometer. Prior to the sorption measurements, sample was heated at 200 °C under vacuum conditions for 3 h. Surface area was calculated by using Brunauer–Emmett–Teller (BET) theory method. The total pore volume (Vt) was measured from the amount of adsorbed nitrogen (at *P*/*P*o = *ca*. 0.99). The wettability of PG samples was characterized by using a contact angle goniometer. Images of each droplet were captured on a digital camera and the contact angle measurements were based on the PolyPro software package.

### Removal of heavy metal and other contaminant ions

All of the chemicals used in the experiments were of the highest purity commercially available and were obtained from sigma Aldrich (detail is given in SI). Three stock solutions of 1000 mg/L arsenic, fluoride and nitrate were prepared by mixing appropriate amounts of sodium (meta) arsenite, sodium fluoride and potassium nitrate salt respectively in distilled water. Standard working solutions at the required concentrations with 130, 150 and 200 mg/l of arsenic, fluoride and nitrate were again prepared by diluting stock solution. 10 mg of PG was added to 25 ml of As (III), fluoride and nitrate working solutions of 130, 150 and 200 mg/l respectively, and the suspension was stirred and separated by filtration through a 0.2 micron membrane filter over different time scale from 0–60 mins. At these times, UV–vis absorption spectra were recorded at 280, 464 and 410 nm to monitor the adsorption processes of arsenic, fluoride and nitrate respectively. To verify the adsorption of fluoride, a fluoride kit was also used. The adsorption isotherms of arsenic on the PG were shown to the best fit to pseudo-first and pseudo-second order kinetic models and an intra-particle diffusion model with the experimental data of this study.

Linear transformations of pseudo-first order and pseudo-second order kinetic models^37^ are, respectively,

1 $$\mathrm{ln}({q}_{e}-{q}_{t})=\,\mathrm{ln}({q}_{e})-{k}_{1}t,$$

and

2 $$\frac{t}{{q}_{t}}=\frac{1}{{k}_{2}{q}_{e}^{2}}+\frac{t}{{q}_{e}},$$

where *t*, *q*_e_, q_t_, *k*_1_ and *k*_2_ represent time, amount of arsenic uptake per unit mass of adsorbent at a particular time, pseudo-first and -second order rate constants, respectively. The values of *q*_e_, *k*_1_ and *k*_2_ were calculated from the slopes of their respective graphs. In addition to these models, Fick’s second law was used to find out if intraparticle diffusion is a rate-controlling step during the adsorption experiment^37,38^:

3 $${q}_{t}={k}_{id}\sqrt{t}+I,$$

where *I* represents the boundary layer effect (a large value corresponds to a larger boundary layer thickness^37,39^) and *k*_id_ is the intraparticle rate constant. The adsorption capacity, *q*_e_, was calculated (as a percentage) using *q*_e_ = *C*_o_-*C*_f_/*C*_o _× 100, where *C*_o_ (mg l^−1^) is solute concentration at equilibrium, and *C*_f_ is the maximum amount of solute adsorbed corresponding to monolayer exposure. To evaluate the stability and reusability of PG, regeneration cycles were repeated five times. 0.2 M HCl solution was used as the desorption agent to recover As (III), fluoride and nitrate from the absorbed PG. The regenerated adsorbent was used for subsequent adsorption cycles under similar reaction conditions as carried out with fresh PG. The initial pH of stock solution was adjusted to neutral (pH 7) using NaOH or HCl solutions. All tests were conducted in triplicate and the average values were used for data analysis. The pH of the solution was maintained at neutral level. We measured the pH of the solution after treatment and found no change in the pH.

### Oil sorption experiment

The oil sorption capacity was achieved and measured by following Standard Test Method for adsorbent performance (ASTMF726–99). Five grades of oils were used in this study; vegetable, engine, pump, used engine and used pump oil. For oil sorption tests, oils were poured into petri dishes. The absorbent was pre-weighed and then weighed again instantly after the experiment. PG were squeezed gently to remove and drained for about 15 mins. 10 mg of PG were dipped in oil and distilled water (50 ml) mixture during this experiment. PG were dropped in a portable folded porous sheet and were removed after a given immersion time before being weighed. To assess the reusability/recyclability and regeneration of the used absorbent, PG were heated up to the boiling point of adsorbate to remove the oil prior to the next round of adsorption test. The regenerated absorbent was used for subsequent sorption cycles under similar conditions as carried out with fresh PG. Each experiment was repeated three times independently and average values were taken. The sorption capacity (*Q*) was determined using the weight (*wt*) of adsorbent before and after the experiment:

4 $$Q=\frac{(w{t}_{{\rm{after}}}-w{t}_{{\rm{before}}})}{w{t}_{{\rm{before}}}}$$

### Organic dyes adsorption experiments

Stock solutions of dyes were prepared by dissolving precisely weighted amounts of methylene blue (MB) and rhodamine b (RB) in distilled water. Working solutions at desired concentrations were prepared by serially dissolving more water in stock solution.10 mg of PG were added to 25 ml of RB and MB solution (of concentration 150 mg l^−1^) and continuously stirred. UV–vis absorption spectrophotometer was used to record their respective spectra at different time intervals to monitor the adsorption at 535 and 496 nm respectively. The adsorption isotherms are fitted by the pseudo first order, second order kinetic and intraparticle adsorption models as described above^37–39^. Regeneration cycles were repeated five times. The adsorbed dyes were eliminated from the obtained adsorbent by heating at 400–450 °C in air for 2–3 h. The regenerated adsorbent was used for subsequent adsorption cycles under similar conditions as carried out with fresh PG.

## Results and Discussion

### Basic characterization

**Figure 1:**
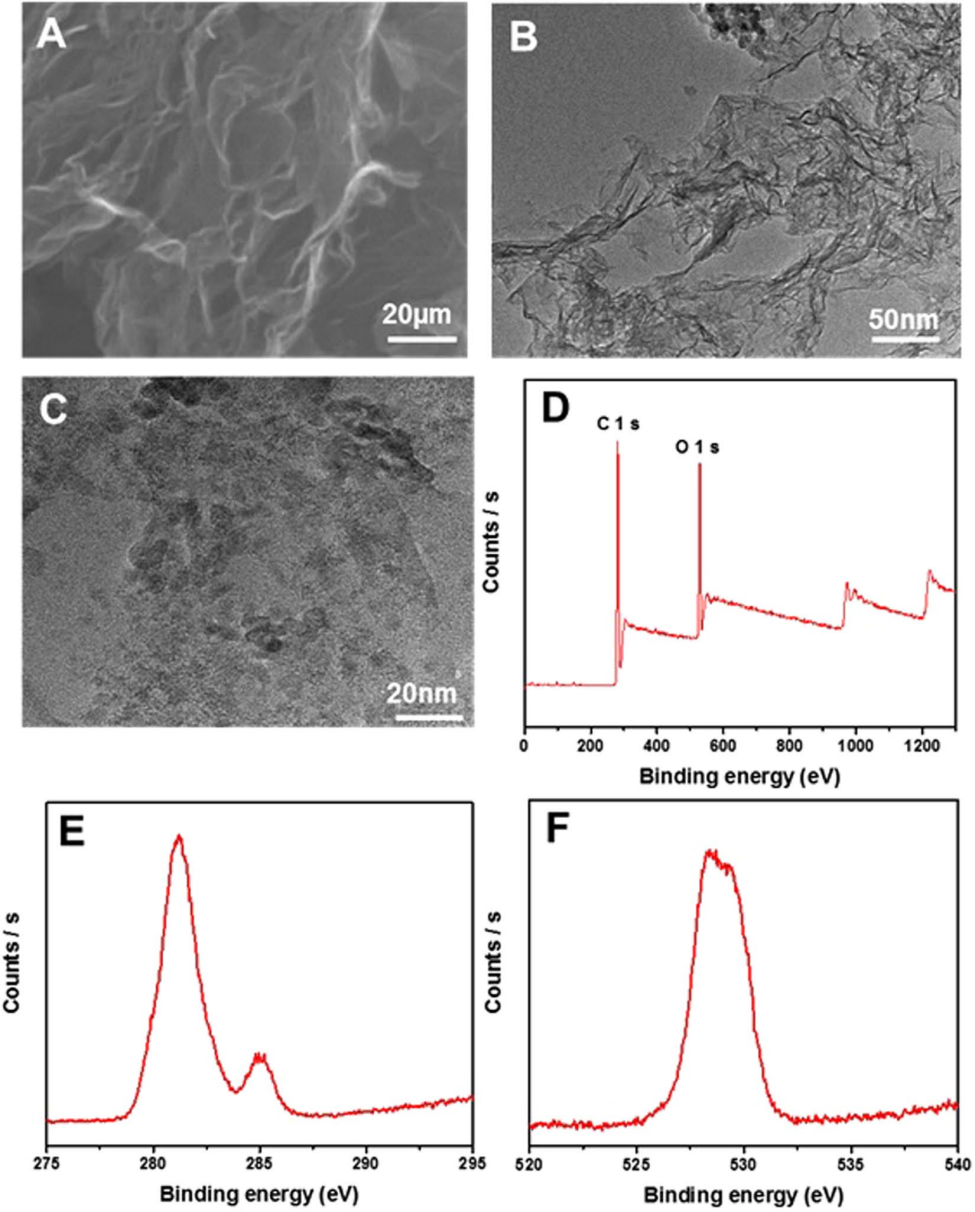
Microstructure and XPS survey of as-prepared PG. (**A**) Low-magnification SEM images of as-prepared PG. (**B**,**C**) TEM images of a representative PG showing holes in the nanosheet. (**D**) XPS survey spectra of PG. (**E**) high resolution C 1 s spectra of, and (**F**) high resolution O 1 s spectra of PG.

Figure 1 shows surface morphology of as-prepared PG. Scanning electron micrographs enable the visualization of wrinkles and corrugations in the graphene sheets^40^, and induce the formation of nano-sized channels or pores on the surface (Fig. 1A). As seen in Fig. 1A, the PG had an irregular, folded structure with sheets entangled with each other. This is further seen in the high-resolution transmission electron micrographs shown in Fig. 1(B,C). These images show folded edges of the flakes with few, irregularly stacked layers^41^ and the formation of pores over the sheets. The SEM images of graphite flakes and GO were shown in Figs S2 and S3. Furthermore, XPS study of the PG revealed that the C 1 s and O 1 s peaks appeared at ~281.18 eV and 528.77 eV, respectively (see Fig. 1D). High-resolution C 1 s and O 1 s scans were carried out in order to investigate the nature of functional groups. Figure 1(E,F) presents the deconvoluted C1s and O 1 s XPS spectra. The intensity of the C 1s spectra corresponding to sp^2^-hydridized carbon revealed the reduction of GO, thermal treatment of rGO and the restoration of graphene sheets^28^. PG (Fig. 1D) mainly was observed to have a peak at around 284.7 eV corresponding to the C=C network due to the sp^2^-carbon, which reveals the predominantly graphitic nature of carbon^42^. Deconvolution of the O 1 s spectra was observed to designate into two peaks, centred at 528.7 and 523.05 eV, which arose mainly from the oxygen containing functional groups (carboxylic, epoxy, carbonyl and hydroxide groups) at the surface of the basal plane of graphene^43^. To further clarify the growth process of the PG structure, the raw material and intermediate product were investigated by XRD, FTIR and Raman spectroscopy.

**Figure 2:**
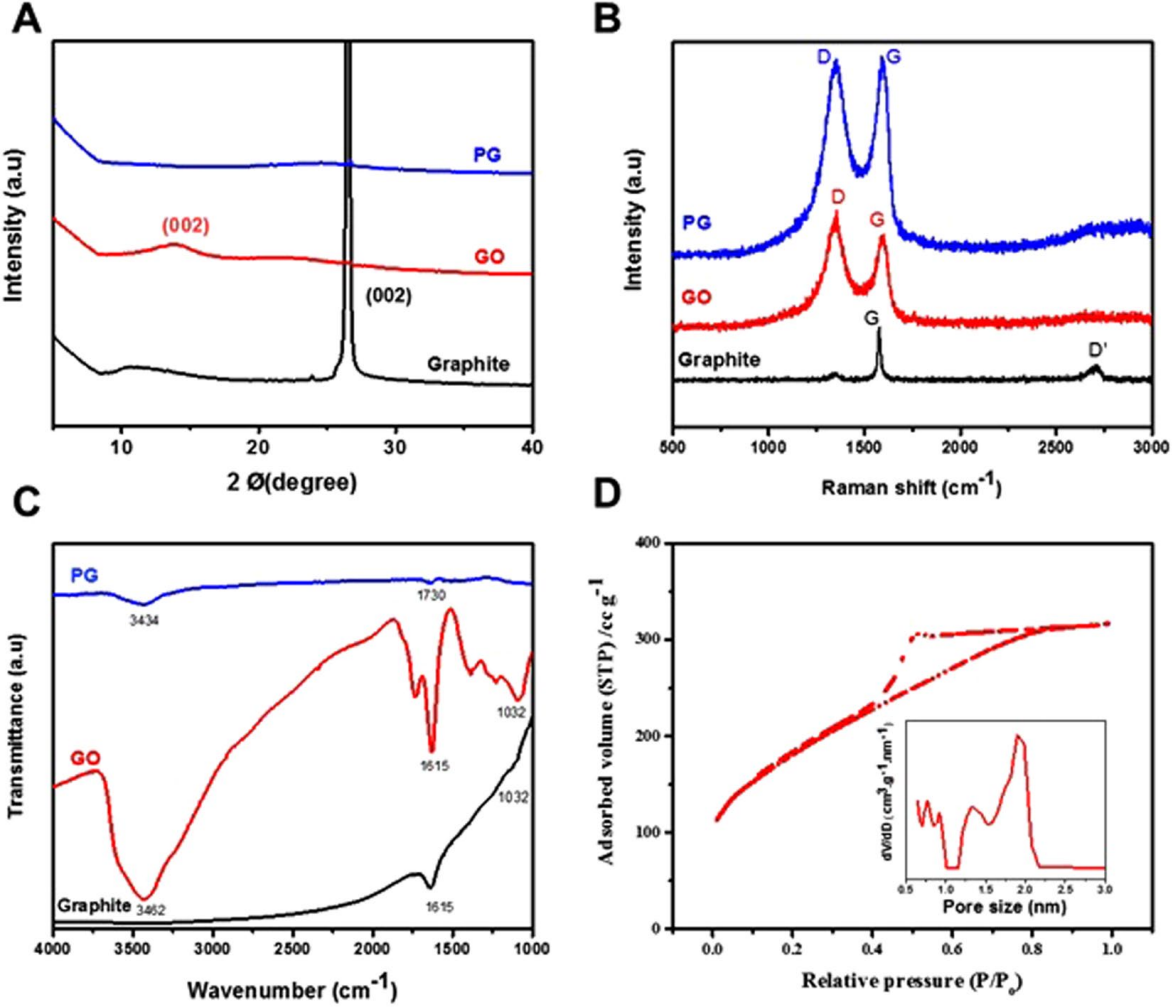
Basic characterization of PG. (**A**) X-ray diffraction, (**B**) Raman spectroscopy, and (**C**) Fourier transform Infrared spectroscopy of graphite flakes (black), GO (red) and PG (blue). (**D**) Measurement of specific surface area. The pore size distribution of PG was calculated using Barrett–Joyner–Halenda method presented in Fig. 2D as an inset at 77 K, pore size 1–4 nm.

A comparison of the XRD patterns of graphite flakes, GO and PG reveals that the (002) peak of PG was broadened and had a markedly reduced intensity, indicating that they were composed of single-layer graphene sheets (Fig. 2A). The introduction of pores on graphene sheets resulted in a change of the D/G Raman peak intensity ratio (Fig. 2B)^44,45^. For graphene-based materials, the Raman G-peak (*ca*. 1590 cm^−1^) corresponded to the sp^2^-hybridized hexagonal lattice of carbon atoms, and the D-peak (*ca*. 1350 cm^−1^) was indicative of sp^3^-hybridized carbon atoms in the lattice structure, which was categorised as defects on the edges of the graphene. In addition, the coexistence of D-peak revealed the sp^2^ hybridized carbon atoms which were slightly reduced as a result of oxidation. However, as indicated in Fig. 2B, as-prepared PG had a higher D/G intensity ratio than graphite and GO, correlating to the reduction in the average size of the sp^2^ domains after the reduction of exfoliated GO. The increased amount of sp^3^-hybrdized carbon atoms indicated the activation of nanosheets for the formation of pores and edges in the nanosheets^46,47^. The FTIR peak of GO was centred at about 1615 cm^–1^, which was shifted to the absorption peak of a carbonyl group at about 1730 cm^–1^ for PG, indicating that the thermal treatment of rGO left more edged carbon atoms in the form of oxygen containing groups (Fig. 2C). To further analyse and quantify the pore structure, nitrogen adsorption–desorption isotherms for the PG sample were determined. Figure 2(D) shows the isotherms and Barrett–Joyner–Halenda pore size distributions of PG. According to the IUPAC classification, the nitrogen adsorption–desorption isotherm curves of these samples exhibited a type IV with a H3 hysteresis loop, which is a characteristic feature of mesopores^41,48^. Furthermore, the adsorption segment of the nitrogen isotherms at *P*/*P*_0_ displayed a steady increase, suggesting the formation of large mesopores and small macropores^41,49^ with the average pore size in the PG calculated as 3–5 nm. The parameters of the samples obtained from nitrogen desorption isotherms are shown in SI Table S1. The BET surface area of PG was calculated as *ca*. 653 m^2^g^−1^, which was significantly higher than that of rGO derived PG (ca. 82.76 m^2^g^−1^) and that of porous rGO reported previously (62 m^2^/g^10^, 31 m^2^/g^50^, 134 m^2^/g^51^, 372 m^2^/g^52^.). The increased pore volume of PG (ca. 0.226 cm^3^g^−1^) compared to porous rGO^50–52^ can be ascribed to the pores induced in the thin-layered sheets of heat-treated rGO. The porous morphology and higher SSA are considered to provide more active adsorption sites for the electrostatic reaction leading to an enhanced adsorption uptake.

**Figure 3:**
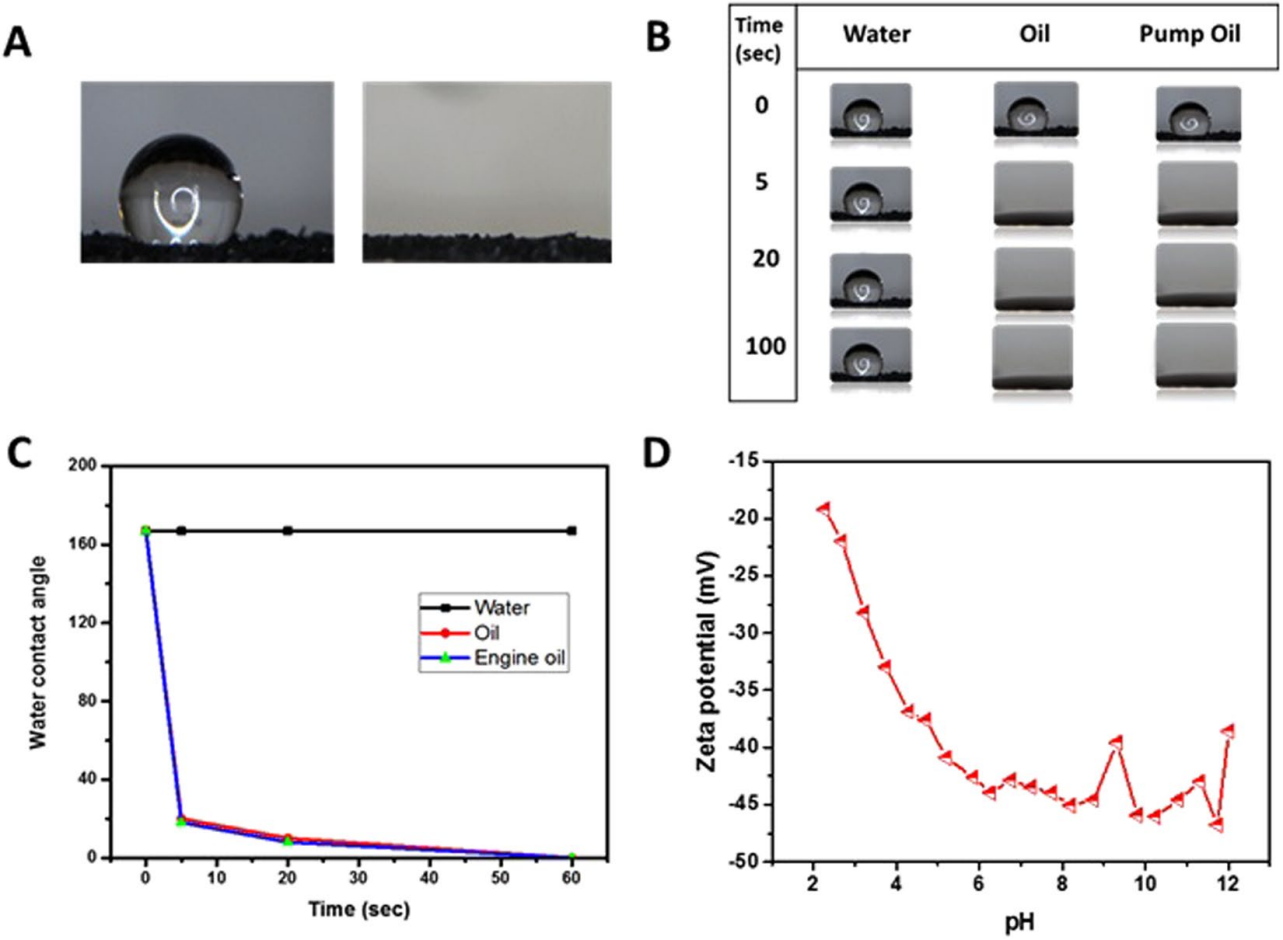
Surface properties of PG. (**A**) Photograph showing a water droplet remaining on PG (left) and disappearance of a droplet of oil (right) after being dropped on PG. (**B**,**C**) Comparison of contact angles for water and different types of oil on PG as a function of time.

Specific characteristics of the nanostructured morphology have a great impact on the wettability of a surface^53^. The wettability of PG surface was studied by water contact angle measurements, and the results are given in Fig. 3(A–C). The PG was found to be superhydrophobic, with water droplet contact angles of 160° formed within 60 seconds (Fig. 3A). In contrast, oils were found to be fully absorbed on PG after 30 s (Fig. 3B), with contact angles of less than 5°, demonstrating that the PG were superoleophilic. The extent of the hydrophobicity of graphene depends on the ethyl functional groups on its surface. The wetting transparencies can be engineered by altering the functional groups, specific surface area, surface roughness and wrinkling network of graphene sheets^54^. The stabilization mechanism and electrostatic interactions are equally important factors that prevent graphene sheets from aggregating in an aqueous solution. The carboxyl and hydroxyl functional groups on the surface of PG indicated that these groups were not reduced by hydrazine^55,56^, as confirmed by the FTIR analysis (see Fig. 2B).

### Removal of heavy metal and other contaminant ions

**Figure 4:**
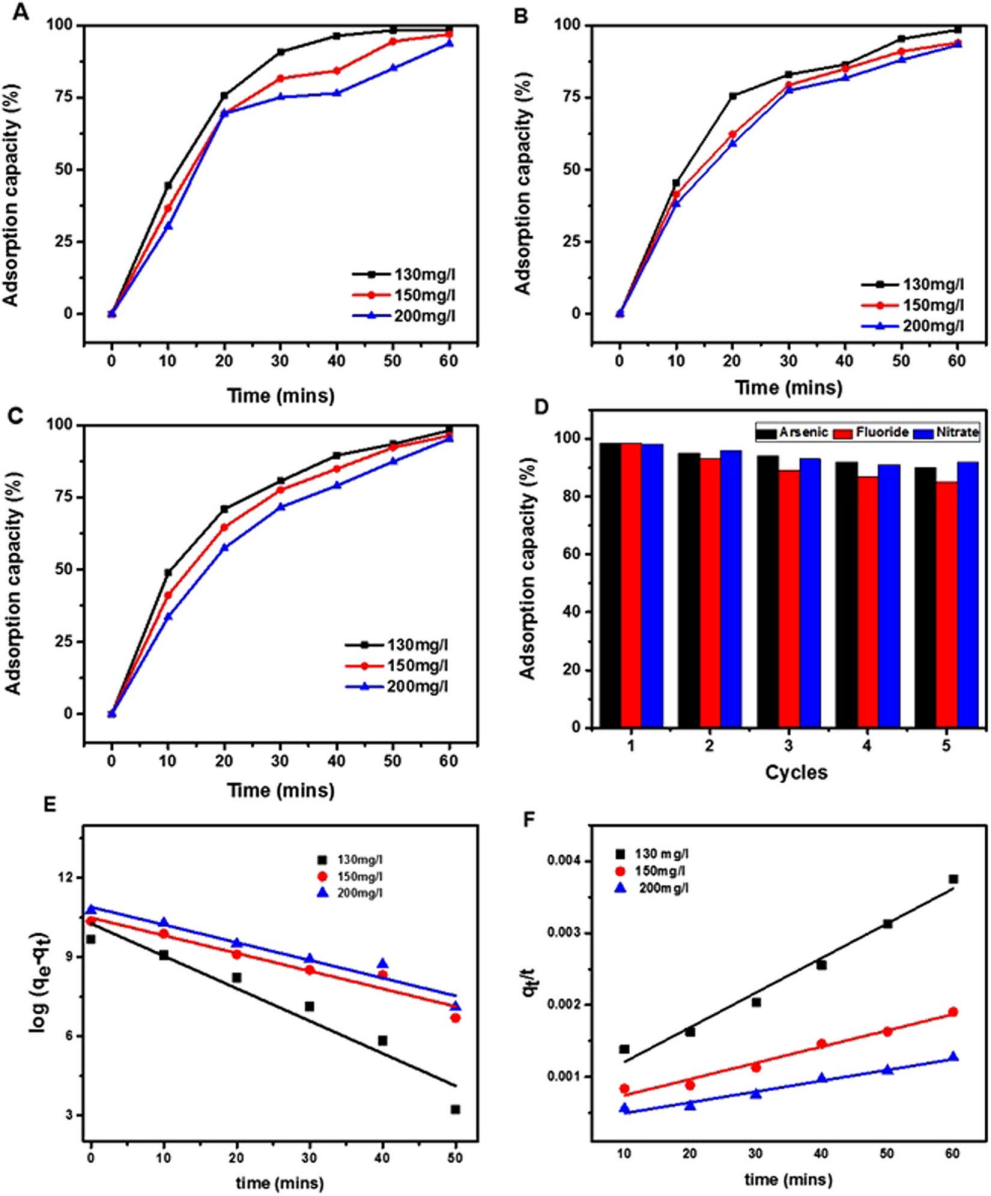
Contaminant ions adsorption properties. The time dependence of adsorption capacity of arsenic (**A**), fluoride (**B**) and nitrate (**C**) by PG at three different concentrations of 130 mg/l, 150 mg/l and 200 mg/l. (**D**) Recyclability of PG for the removal of arsenic, fluoride and nitrate. (**E**) Pseudo-first and (**f**) -second order models was found to fit the adsorption of arsenic (100 mg/l) on PG.

Arsenic, fluoride and nitrate are some of the most prominent contaminants toxic to living species^57^. Batch adsorption studies were conducted to measure the adsorption properties of these ions onto PG with Fig. 4 showing that an increase in adsorption capacity occurred for all ions over a 60 minute time period, reaching a maximum capacity of 99%. The capacity was found to be slightly greater for low concentrations of PG with both kinetic models showing a good agreement with the experimental data (Fig. 4E and F). However, by quantitatively comparing the adsorption capacity, *q*_*e*_, calculated from equations 1 and 2 with the experimental value, it was demonstrated that arsenic adsorption followed the pseudo-first order kinetic model, based on which the parameters were calculated and are given in SI Table S2. PG exhibited significantly higher arsenic adsorption capacity than the conventional adsorbents, such as activated carbon^58^, carbon nanotubes^59^, three dimensional graphene^60^, and 2D GO sheets^61^. The reason for this was likely to be that PG had significantly more active adsorptive sites as a result of the higher SSA and functional groups. The hydrophobicity of PG indicated the diffusion and incorporation of metal ions into their surface and internal regions keeping water away from those places. The adsorption kinetics of As (III) was very rapid initially, with about 80% of total As (III) ions being removed within 30 mins (Fig. 4A).

Figure 4B and C show the adsorption capabilities of PG for the elimination of fluoride and nitrate. The adsorption capacity of fluoride (Fig. 4B) followed a similar trend to that of As (III), and was much higher than that reported by Gupta *et al*.^62^. When fluoride adsorption was tested using a fluoride kit, complete elimination of fluoride from aqueous solution was achieved, as shown in Fig. S4 in SI. The adsorption capability of as-prepared PG for all the tested ions can be ordered as As > Fluoride > Nitrate. The prominent single atom thick sp^2^ hybridized feature of graphene was responsible for its appreciable and rapid interaction with arsenic ions. Electrostatic interaction between PG and contaminant was also responsible for the fast adsorption kinetics of arsenic. The capacity for regeneration and reusability of PG for the removal of these micro pollutants was also examined. The adsorbent can subsequently be recycled after desorption of PG. Figure 4(D) shows the recyclability of PG in terms of the adsorption capacity of these metal ions. It was observed that the adsorption capabilities of regenerated PG remained considerably high, > 80% after 5 cycles, suggesting an environmentally friendly recyclability of PG for the decontamination of a variety of target contaminant ions.

### Sorption of Oil

**Figure 5:**
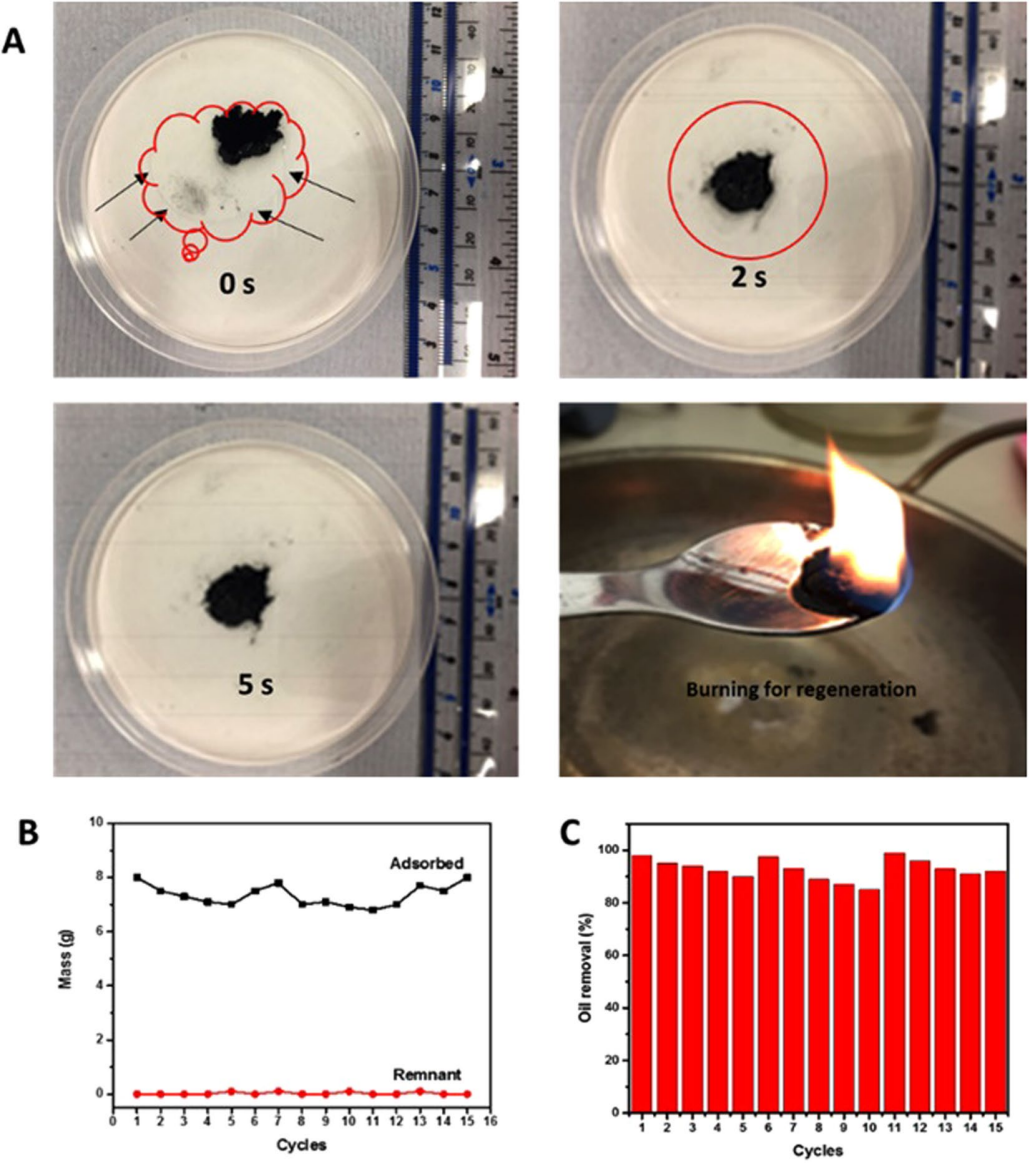
Oil absorption properties. (**A**) Pictures showing the vegetable oil absorption refining process into PG as a function of time. The edge of the oil drop is indicated by the red line. The bottom right photograph shows the regeneration process. (**B**) Comparison of the weight of a PG sample before and after adsorption. (**C**) Absorption recyclability of PG for oil.

As-prepared PG were used as a sorbent to examine their sorption efficiency for a variety of oils. Figure 5(A) shows that, upon their immersion, the oils were immediately absorbed and completely taken up within just 20 seconds. They exhibited oil absorbing capacities over a range between 54–165 times their own weight (Fig. 5B), indicating their superior oil-sorption capacity to other absorbents previously reported^63^. In a further test, as-prepared PG samples were wrapped in a combined oil and water system in a beaker (see SI Fig. S5). It was observed that they took up all the oil, and the oil uptake capacity from the mixture was very close to that from pure oil. Figure 5(C) illustrates the recyclability of PG in the oil uptake process. In the second cycle, the sorption capacity decreased, which was most likely due to an incomplete regeneration process: repeated cycling showed that the capacity was overall consistent and above 90% even after 15 cycles. All of these results indicated that as-prepared PG can be used as an effective, simple filter for the decontamination of large quantities of oils directly from wastewater.

### Adsorption of Organic dyes

**Figure 6:**
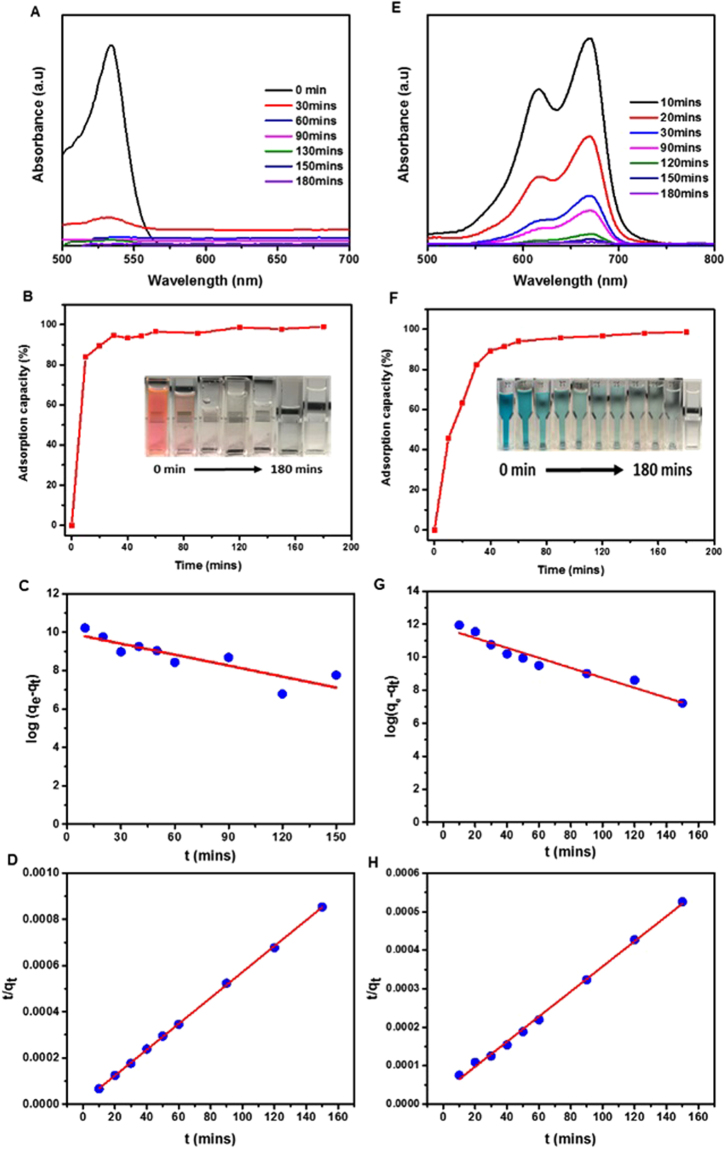
Dyes adsorption properties. Adsorption of RB (**A**–**D**) and MB (**E**–**H**) in aqueous solutions by PG is shown. (**A**,**E**) UV–vis absorption spectra shown for different times after adsorption. (**B**,**F**) Adsorption rates. The insets are corresponding photographs showing colour change of RB and MB from pink and blue respectively to transparent within 180 mins. Pseudo-first (**C**,**G**) and -second order (**D**,**H**) kinetic models fitting to the data.

It was found that RB and MB are primary toxic pollutants in water resources^64^. The adsorption kinetics of the former were evaluated based on UV–vis absorption tests (Fig. 6A). Figure 6(B) shows that the adsorption of RB was completed within 60 mins with about 99% removal efficiency. It can be seen that the RB absorbance band became weak after 60 mins. The adsorption isotherm, Fig. 6(C,D), can be fitted well by the kinetic models. The value of the adsorption capacity extracted from the pseudo-first order kinetic model was found to agree well with the experimental results, whereas the pseudo-second order value compares poorly. Therefore, in the case of RB, the adsorption followed the first kinetic model. The parameters of these models are listed in SI Table S2. The adsorption capacity was significantly greater than that reported for conventional adsorbents^65–67^. The adsorption capacity of MB has been shown in Fig. 6(D–F). The maximum capacity was measured as 313 mg g^−1^. As in the case of RB, MB adsorption capacity values were also greater than those reported for conventional adsorbents (SI Table S3). Taking these experimental and theoretical conditions into account, it was revealed that the highest value of correlation coefficient, corresponding theoretical and experimental values of *q*_e_ obtained from pseudo-second order kinetics model indicated that both the external and internal mass transfers took place^37^. These results also indicated that the pseudo-second order kinetic model fitted the adsorption curve much better than pseudo-first order kinetic model. The PG were recycled five times after regeneration via heating at 600 °C in air. The adsorption isotherm after the fifth cycle, and the recyclability of both RB and MB are shown in Figs S6 and S7, respectively in SI. Recyclability remained higher than 90% even after the fifth cycle. The fact that as-prepared PG showed much higher adsorbencies of dyes than commercial bulk graphene particles indicated that the porous architecture and high SSA played important roles in the enhancement of adsorption capacity. The adsorption capacities of PG to adsorb contaminated ions, oil and dyes was remarkably high in comparison to those obtained using other similar nanostructure based adsorbents reported recently (SI Table S3), suggesting that the porous nanostructures of graphene prepared here are highly efficient potential materials for the elimination of variety of contaminants from water and wastewater.

### Adsorption mechanism and structural evolution

**Figure 7:**
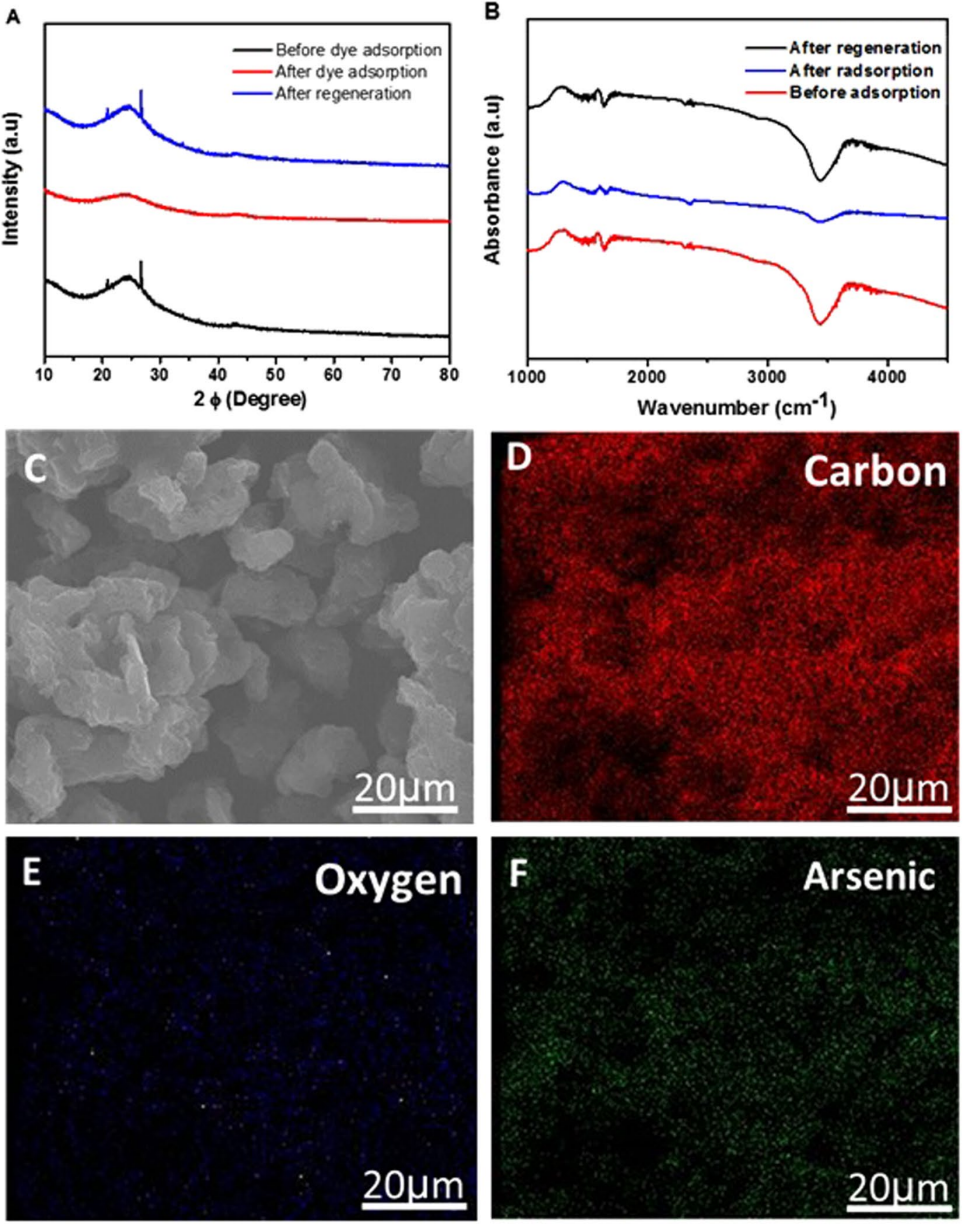
Structural evolution and elemental mapping of PG. (**A**) XRD, and (**B**) FTIR of PG before and after adsorption of dye and also after regeneration. (**C**) SEM image of PG after adsorption. (**D**–**F**) Elemental mapping (C - red; O – blue; Ar- green).

**Figure 8:**
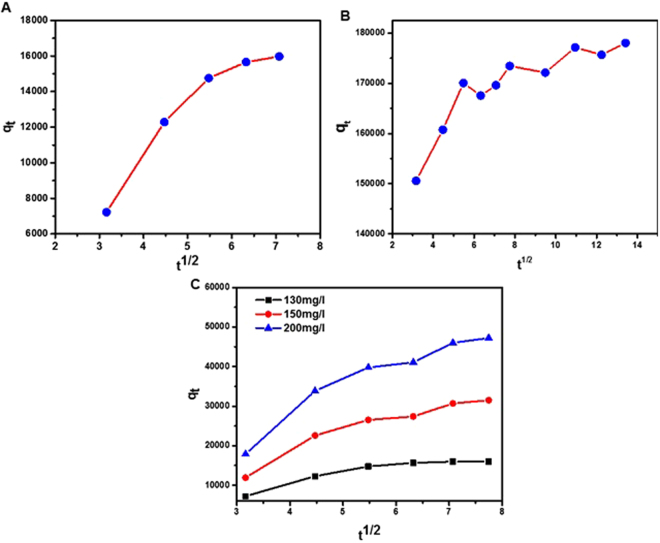
Piecewise linear regression analysis of the adsorption experiments of (**A**) arsenic, (**B**) RB dye and (**C**) MB dye on PG.

To understand the adsorption phenomena, it is necessary to characterize the surface structure of PG before and after adsorption and also after regeneration/release of adsorbates from PG. We used XRD and FTIR to illustrate the structural evolution of binding pollutants on the surface of the PG. Figure 7(A) shows the XRD results. The appearance of the (002) diffraction peak towards lower angles were explained by an increased distance between the (002) planes which was arisen by intercalating the reactants into interlayer space. The surface functional groups before and after adsorption of arsenic on PG were examined by FTIR as shown in Fig. 7B. The peaks at 3434 cm^−1^ (OH), 2362 cm^−1^ (CO_2_) and 1726cm^−1^ (C = O) and 1627cm^−1^ (C = C), clearly shifted before and after adsorption and these functional groups were also remained after regeneration. The band at 3434 cm^−1^ was ascribed to the OH bond of adsorbed water. The band appeared at 2362 cm^−1^ was attributed to CO_2_ stretching vibration, which weakened after the adsorption of arsenic, revealing the exchange between carbonates as well as arsenite^68^. A weak vibration peak at 3434 cm^−1^ indicated the significantly efficient arsenic adsorption onto the surface of PG, and was ascribed to As–O stretching vibration. In order to further reveal the adsorption of arsenic and fluoride, PG samples, before and after adsorption were characterized by elemental mapping. Figure 7C–F shows the results of elemental mapping on PG, providing direct evidence of the adsorption of the contaminant ions. Mapping was carried out for the sample in the case of arsenic (Fig. 7B–D), revealing the presence of traces of carbon, oxygen and arsenic. Fluoride-adsorbed PG samples were also studied and the results are shown in Fig. S8 in SI. Piecewise linear regression kinetics was performed in order to better understand the underlying mechanism of the adsorption process. The three linear segments acquired from linear regression model indicated that adsorption of arsenic, RB and MB on the PG occurred via diffusion of contaminant molecules to the surface of PG (Fig. 8). This mass transfer and diffusion occurred through the surface layer, pores and internal surface of the graphene pores. The liquid–solid surface interaction can be broadly ascribed to the energetics of immersion of PG in a liquid as PG facilitated rapid diffusion. The Raman spectra shown in SI Figure 9 show very small difference in Raman shift after burning the adsorbent. The small shift in Raman D band (*ca*. 1360 cm^−1^) indicated the intercalation of oil molecules in the inter-layer space of graphene, which is indicative of sp^3^-hybridized carbon atoms associated with the trapped oil in the lattice structure.

Based on the above analysis, we find five distinct reasons for the high adsorption capacities of the PG:

1. The synthesis of PG produces several types of active sites.
2. Surface complexation occurs between contaminants and the surface hydroxyl groups.
3. Co-exchanges occur between arsenite and fluoride anions as well as the surface hydroxyl and carboxyl groups. Arsenic and fluoride can react with PG adsorbent as given in equations (5–8*):*
  5 $$PG-O{H}_{2}^{+}+{F}^{-}\to PG-OH-{F}^{-}+O{H}^{-}$$
  6 $$PG-COO{H}^{+}+{F}^{-}\to PG-COO{H}^{+}-{F}^{-}$$
  7 $$PG-O{H}_{2}^{+}+{H}_{2}As{O}_{4}^{-}\to PG-COO{H}_{2}^{+}-{H}_{2}As{O}_{4}^{-}$$
  8 $$PG-COO{H}_{2}^{+}+{H}_{2}As{O}_{4}^{-}\to PG-COO{H}_{2}^{+}-{H}_{2}As{O}_{4}^{-}$$
  where PG is representing porous graphene. Reactive functional groups react quite readily with most of the organic pollutants and also cleave the bonds in organic molecules. Free radical generation in adsorbents also play a vital role in adsorption. As a large number of free radicals may provide more active and chemically reactive sites for chemisorption where organic molecules or metal ions might extensively be adsorbed. Free radicals contain a strong initiation source of adsorption and can overwhelm the majority of the organic compounds and contaminated ions.
4. The abundant channels (porous architecture) on the PG surface are favourable to the adsorption of contaminants having different sizes.
5. Surface area introduces stacking sites and physiochemical partition to adsorb molecules because of the availability of significant transformation sites. Generally, the affinity in the adsorption process between adsorbent and adsorbate depends on the high surface area.

## Conclusion

In summary, we investigated facile, scalable and novel synthesis of PG and its application for the treatment/removal of pollutants present in water and wastewater. As-prepared PG material exhibited highly selective absorption and adsorption capacities of about 99% for a wide range of ecologically important pollutants, including heavy metal ion (arsenic) and other contaminated ions (fluoride and nitrate), oils (vegetable, engine, pump, used engine and used pump oil) and dyes (methylene blue and rhodamine B). The dominant mechanisms of surface complexation and co-exchanges with hydroxyl and carboxyl groups of graphene nanosheets were revealed. PG was nontoxic and environmentally friendly, and was demonstrated to not only have highly efficient adsorptive properties but also have superior regeneration and cycling efficiency (above 90% after 5 cycles). Therefore, the application of developed PG materials could contribute towards efficient water/wastewater treatment, reduce pollution load and improve the access to safe drinking water in areas where groundwater is contaminated. The conventional energy intensive treatment processes employed are expensive and unsustainable and show low efficiency in contaminant removal. An improved understanding of complex interactions and interferences from contaminants to the treatment of water and wastewaters under controlled conditions can provide insights into pollutant specific treatment kinetics and support the development of compact optimal treatment strategies.

## Electronic supplementary material

Supplementary information
