# Supplementary material for: A facile synthesis of porous graphene for efficient water and wastewater treatment
Source: Sci Rep. 2018 Jan 29;8:1817. doi: 10.1038/s41598-018-19978-8 (PMC5788977; doi:10.1038/s41598-018-19978-8)
Supplement: Supplementary file 1 — Supplementary information [file 41598_2018_19978_MOESM1_ESM.pdf]

Supplementary information

A facile synthesis of porous graphene for efficient water and wastewater treatment

Tanveer A. Tabish, Fayyaz A. Memon*, Diego E. Gomez, David W. Horsell, and Shaowei Zhang*

College of Engineering, Mathematics and Physical Sciences, University of Exeter, Exeter EX4 4QF, United Kingdom

* Address correspondence to f.a.memon@exeter.ac.uk; s.zhang@exeter.ac.uk

Materials and reagents

$NaNO_3$ (Product No. S5506), H_2SO_4 (95.0-98.0%, Product No. 320501), $KMnO_4$ (Product No. 223468), H_2O_2 (30 wt%, Product No. 216763), hydrazine (35 wt%, product No. 309400), sodium fluoride (product No.: 7681-49-4), sodium (meta) arsenite (product No.: S7400), potassium nitrate salt (product No.: 542040) were purchased from Sigma-Aldrich. HCl (36 wt%, product No.: 7647-01-0), was purchased from Acros. Graphite flake (GFs, product No.17346-25) and 0.2 micron membrane filter were purchased from Thermo and Fisher Scientific suppliers. Fluoride kit (Product No.: 90734, test fluorure was purchased from MACHEREY-NAGEL GmbH & Co. KG, Germany).

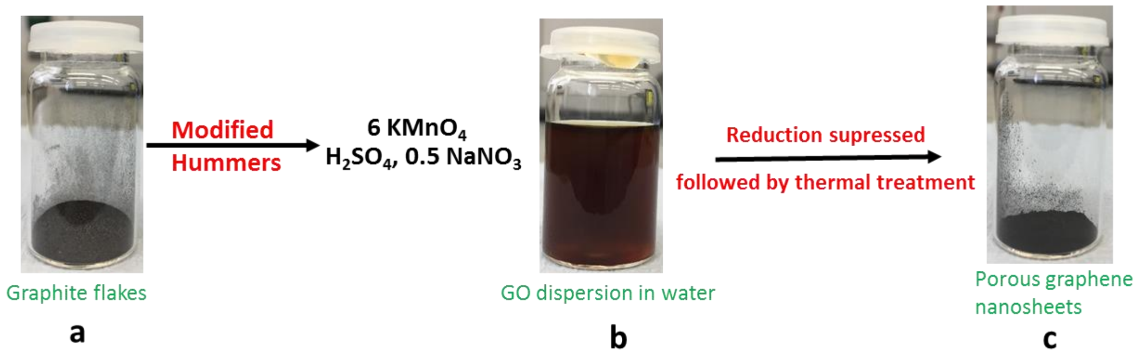

Figure S1: Photographs of the stages in producing PG. (a) Oxidation and exfoliation of graphite in the presence of oxidizing agents. (b) GO dispersed in water prior to reduction. (c) Reduction of GO using hydrazine as reducing agent followed by thermal treatment results in the as-prepared PG in powder form.

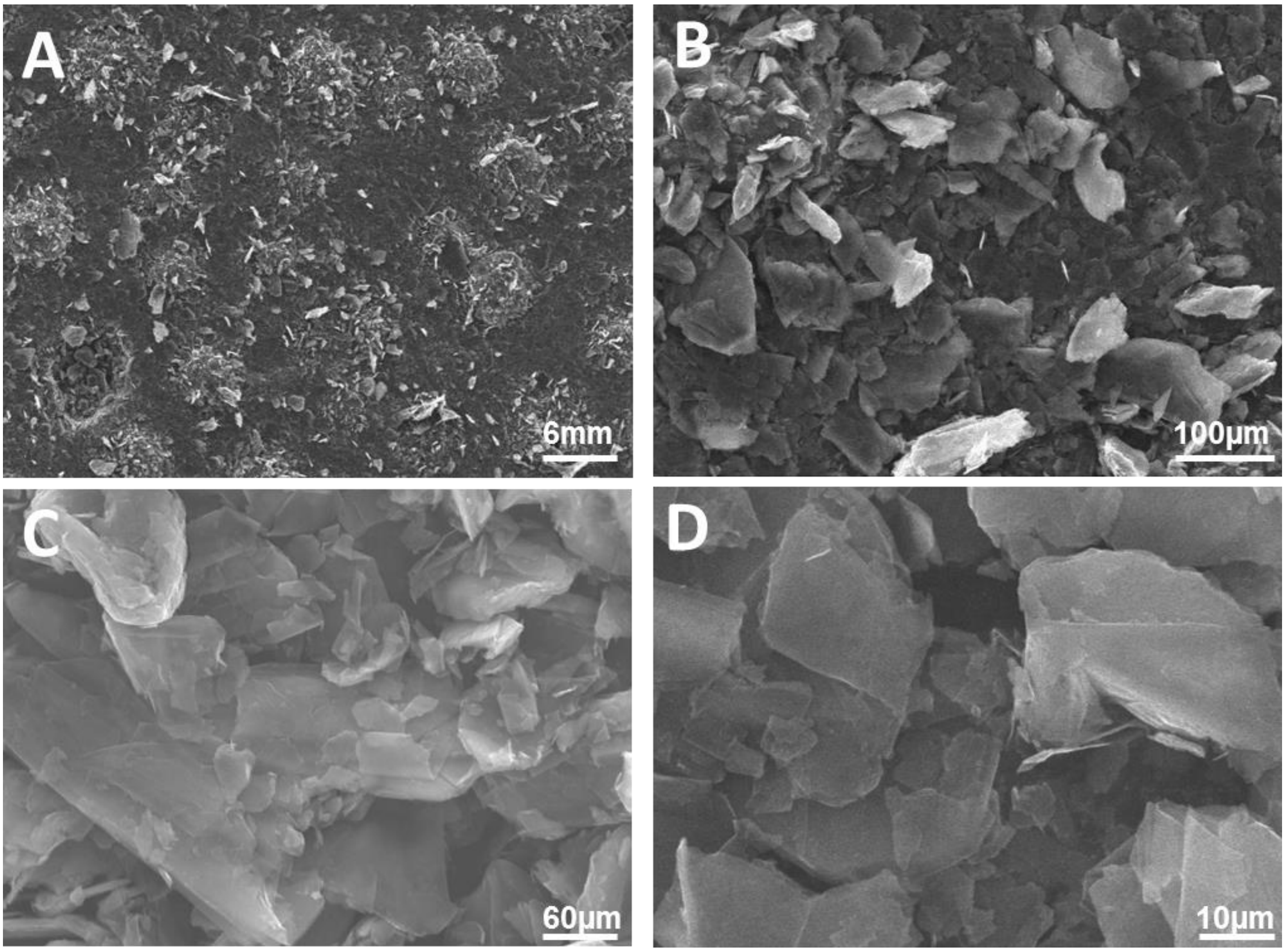

Figure S2: Exfoliation of graphite flakes. (a-d) SEM images of the raw material graphite flakes. Images of the residual graphite sediment, showing many disintegrated and exfoliated graphite flakes.

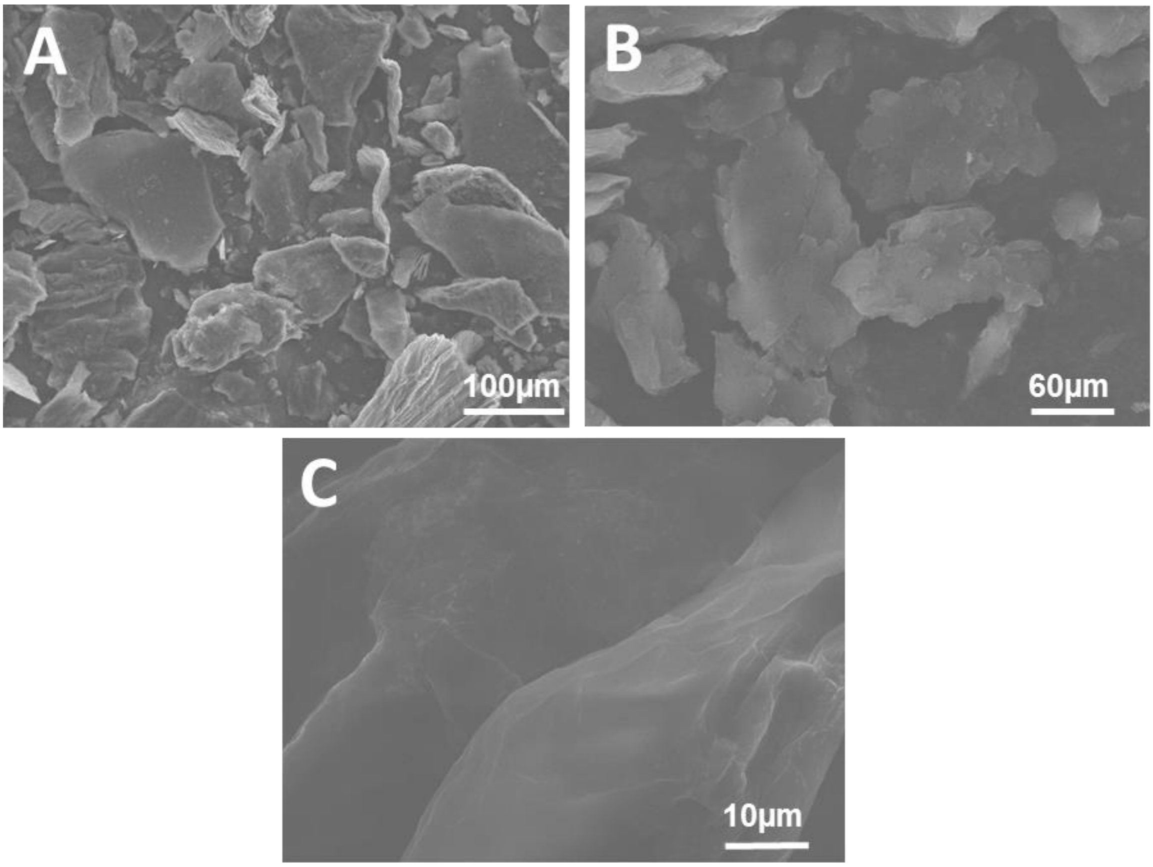

Figure S3: (a-c) SEM images of as-prepared GO. The flaky texture reflects the layered structure of GO.

Table S1: Parameters obtained from nitrogen desorption isotherms.

**SA[a] [m^2g^{-1}]**	**PV[b] [cm^3g^{-1}]**	**PD[c] [nm]**
653 m^2/g	4.904e-01 cc/g	1.5037 nm

a BET surface area.

b Pore volume: DFT desorption cumulative volume of the pores.

c Pore diameter: average pore diameter determined by the BJH method.

Micropore Volume : 0.187 cc/g

Total pore volume = 4.904e-01 cc/g for

Average pore Radius = 1.50370e+01 Å = 1.5037 nm

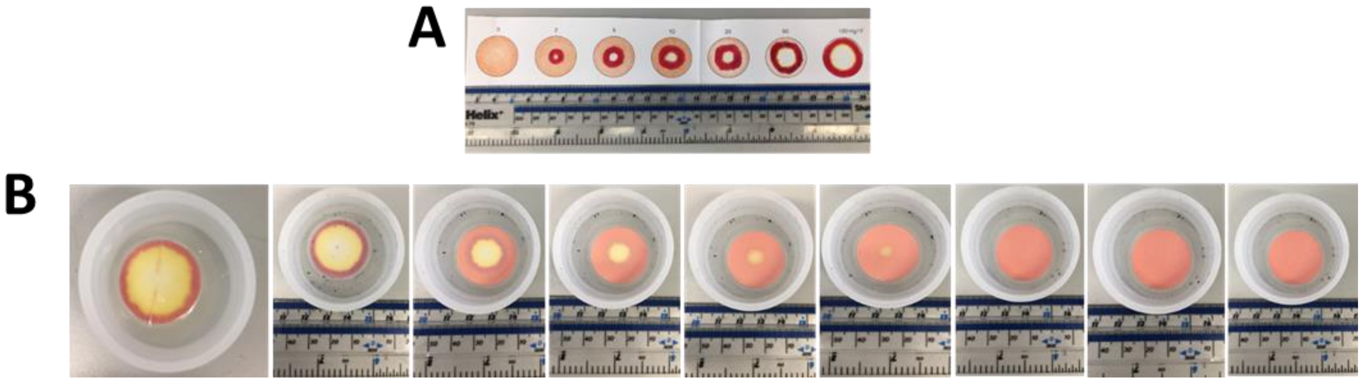

Figure S4: Fluoride kit testing of fluoride removal from drinking water. (a) The standard concentration of fluoride in aqueous solutions using fluoride kit varying from 0, 2, 5, 10, 20, 50, 100mg/l fluoride in water. (b) 100mg/l fluoride sample tested using PG over the exposure time of 5, 10, 15, 20, 30, 60 mins, 24 hrs and 48 hrs. It is found that adsorption process has been completed within 60 mins using PG as adsorbent.

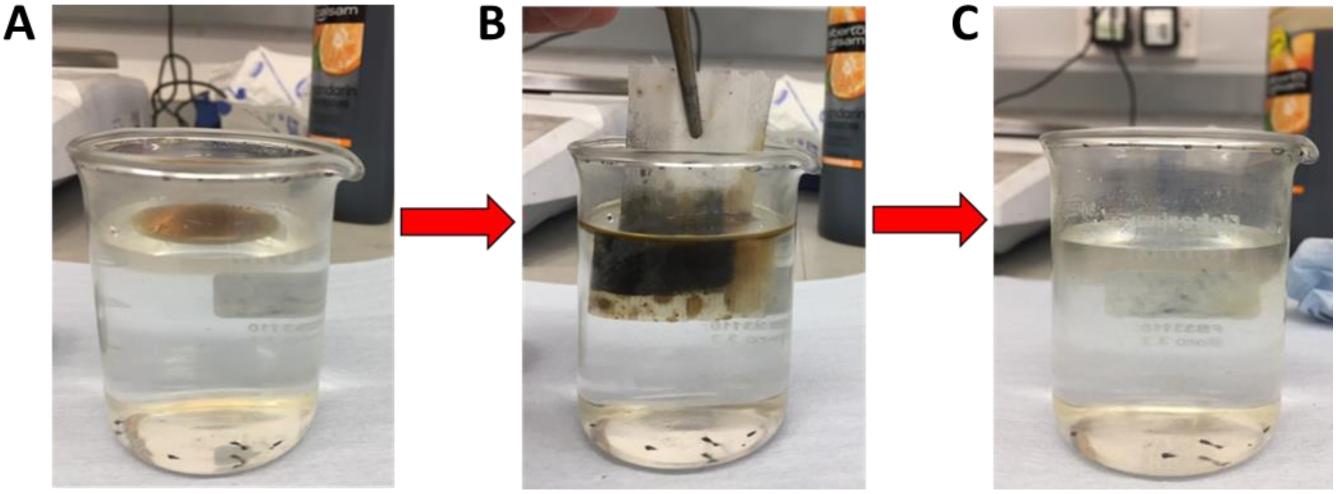

Figure S5: Photographs showing the removal process of pump oil. (a) 1 g oil in 50 ml water, (b) immersion of 2.5 mg PG contained in a porous bag for oil removal, and (c) the oil-absorption after 10 sec.

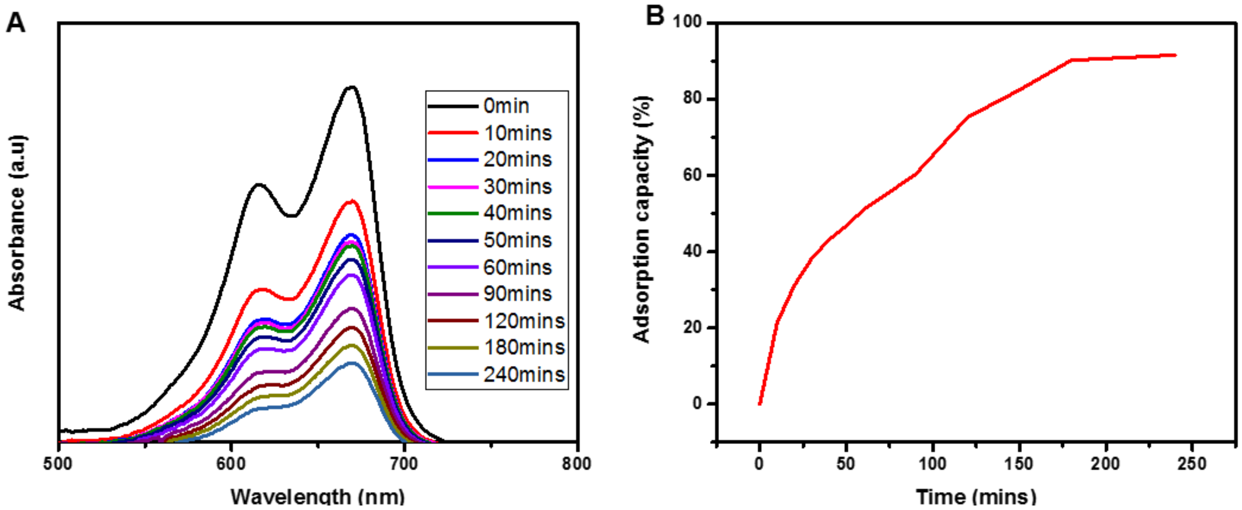

Figure S6: Time evolution of MB adsorption properties of PG after the fifth regeneration cycle. (a) Adsorbance as a function of wavelength. (b) Adsorption capacity. The data show the complete removal of MB within 240 mins.

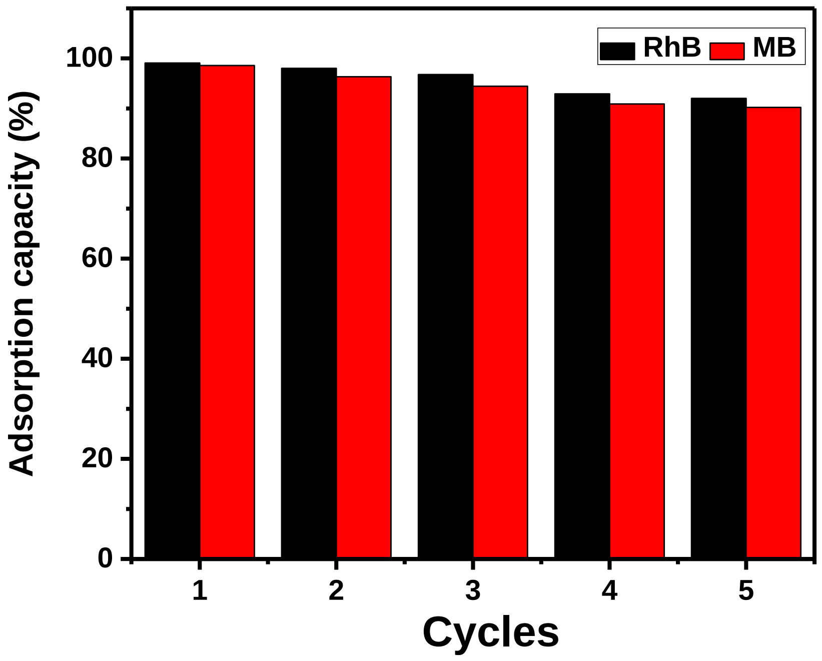

Figure S7: Recyclability of PG for the adsorption of MB and RB.

Table S2: Analysis of the results of adsorption experiments of arsenic, RB and MB on PG

Adsorbate	Order	k_1 / k_2	q_e (mg g^{-1})	y	R^2
Arsenic (130mg/l)	1st order	k_1 = 0.14 min^{-1}	5.4*10^4	-0.1408x + 10.922	0.9473
	2nd order	k_2 = 3.57 *10^{-6} g mg^{-1} min^{-1}	2*10^4	4E-05x + 0.0008	0.9777
Arsenic (150mg/l)	1st order	k_1 = 0.06 min^{-1}	3.6*10^4	-0.0673x + 10.494	0.9429
	2nd order	k_2 = 8.0*10^{-7} g mg^{-1} min^{-1}	5*10^4	2E-05x + 0.0005	0.9747
Arsenic (200mg/l)	1st order	k_1 = 0.067 min^{-1}	5.4*10^4	-0.0673x + 10.9	0.9429
	2nd order	k_2 = 1.3*10^{-5} g mg^{-1} min^{-1}	5*10^4	2E-05x + 0.0003	0.9747
RB	1st order	k_1 = 0.02 min^{-1}	2.2*10^4	-0.019x + 9.9738	0.7677
	2nd order	k_2 = 3.6*10^{-6} g mg^{-1} min^{-1}	16.6*104	6E-06x + 1E-05	0.9998
MB	1st order	k_1= 0.0302 min^{-1}	1.29* 10^5	-0.0302x + 11.773	0.9409
	2nd order	k_2 = 3*10^{-7} g mg $^{-1}$ min^{-1}	3.3*10^5	3E-06x + 3E-05	0.9974

Table S3. Comparison of the adsorption capacities and performance of different contaminants from water between PG and previous reported different adsorbents.

**Adsorbents**	**Surface area** ($m^2\ g^{-1}$)	**Contaminant**	**Adsorption capacity**	**References**
PG	653	Arsenic, fluoride and nitrate	100% in 45-60mins	This work
PG	653	Oil	Above 98 %	This work
PG	653	MB and RhB	100 % in 3 h	This work
Iron loaded activated carbon	-	As	80%	[1]
Carbon nanotubes	110	As	92 %	[2]
Magnetite reduced graphene oxide composites	148 and 117	As	99 %	[3]
Three dimensional graphene	560	As	95 %	[4]
Carbon slurry	629	Fluoride	90 %	[5]
Composites based on activated carbon	-	Nitrate	96.7%	[6]
Activated carbon	119	Nitrate	41.2%	[7]
Activated carbon	-	MB	100% in 4 h	[8]
Carbon	167	MB	97 %	[9]
Graphene oxide	32	MB	98.8%, in 5 h	[10]

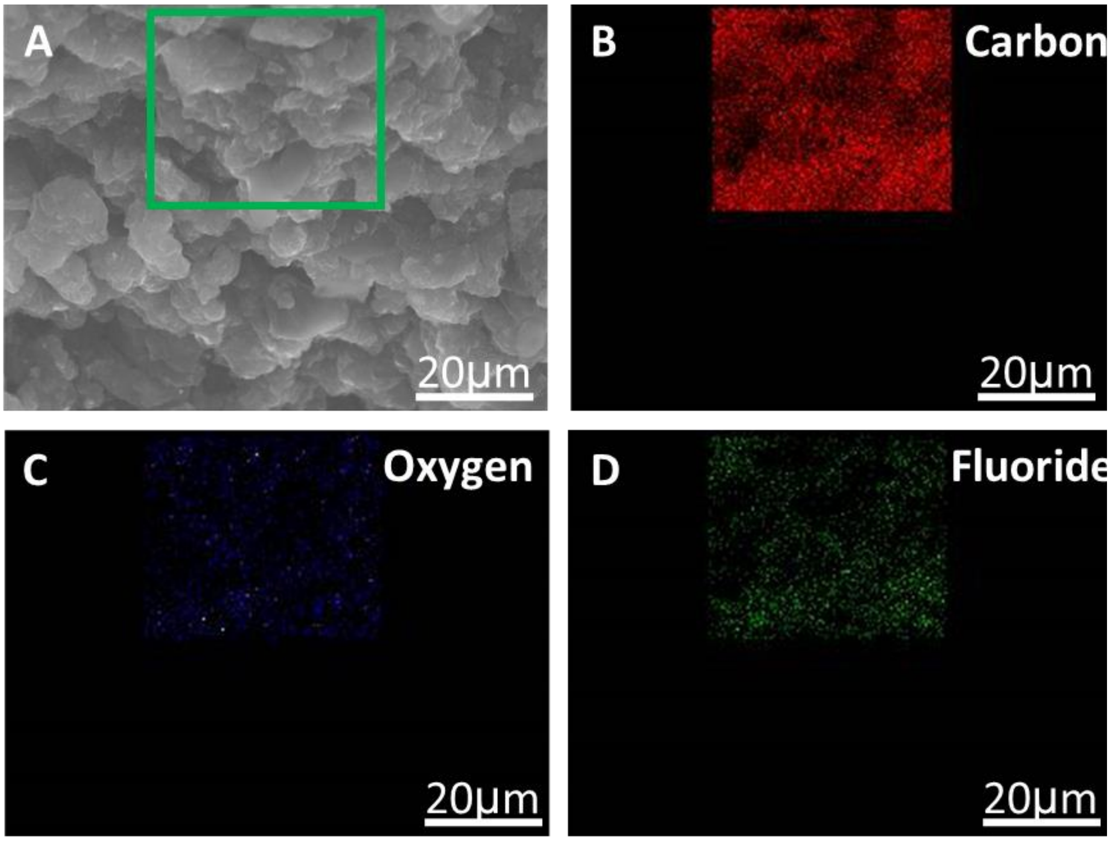

Figure S8: Elemental mapping of fluoride adsorption using an SEM. (a) electron micrograph of PG after adsorption. (b-d) Mapping of carbon, oxygen and fluoride over the area highlighted in (a).

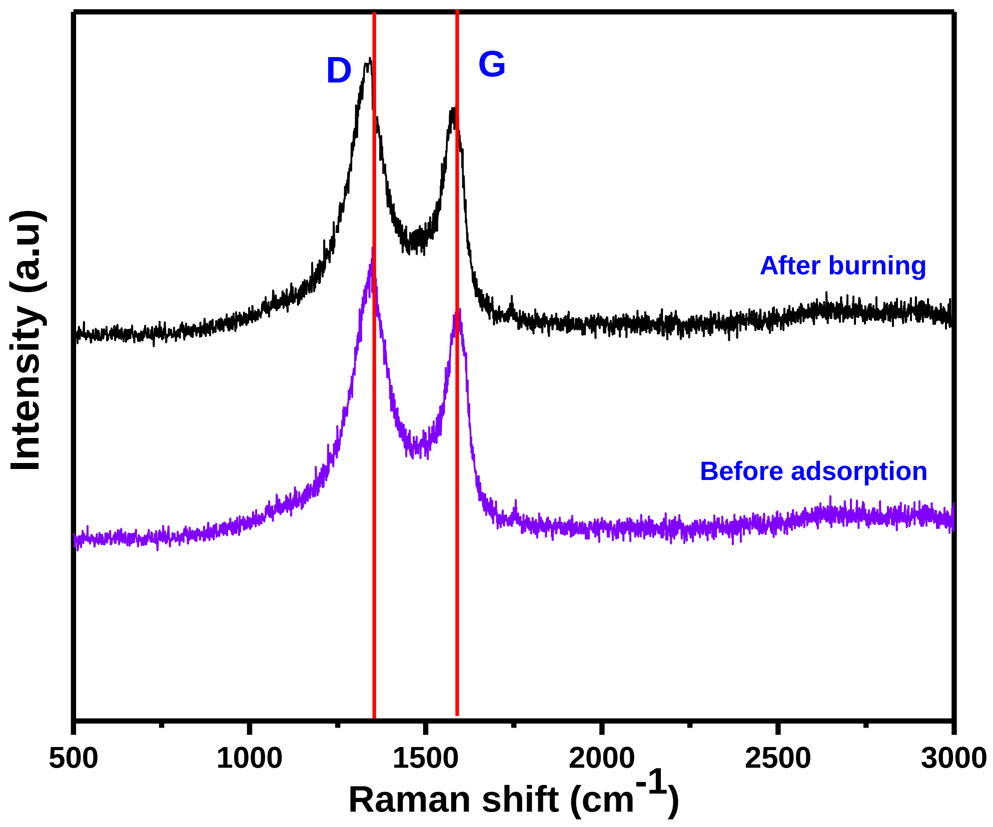

Figure S9: Raman spectroscopy of PG before adsorption and after regenration PG

References:

[1] Chen, W., Parette, R., Zou, J., Cannon, F. S., & Dempsey, B. A. Arsenic removal by iron-modified activated carbon. *Water Res.* ***41***, 1851 (2007)

[2] Ntim, S. A., & Mitra, S. Adsorption of arsenic on multiwall carbon nanotube–zirconia nanohybrid for potential drinking water purification. *J. Colloid. Interf. Sci.**375***, 154 (2012)

[3] Chandra, V., Park, J., Chun, Y., Lee, J. W., Hwang, I. C., & Kim, K. S. Water-dispersible magnetite-reduced graphene oxide composites for arsenic removal. *ACS nano* ***4***, 3979 (2010)

[4] Li, W., Gao, S., Wu, L., Qiu, S., Guo, Y., Geng, X., & Long, M. High-density three-dimension graphene macroscopic objects for high-capacity removal of heavy metal ions. *Sci. Rep.* ***3***, (2013)

[5] Gupta, V. K., Ali, I., & Saini, V. K. Defluoridation of wastewaters using waste carbon slurry. *Water Res.* ***41***, 3307 (2007)

[6] Öztürk, N., & Bektaş, T. E. Nitrate removal from aqueous solution by adsorption onto various materials. *J. Hazard. Mater.**112***, 155 (2004)

[7] Demiral, H., & Gündüzoğlu, G. (2010). Removal of nitrate from aqueous solutions by activated carbon prepared from sugar beet bagasse. *Bioresource. Tecnol.* ***101***, 1675 (2010)

[8] Senthilkumaar, S., Varadarajan, P. R., Porkodi, K., & Subbhuraam, C. V. Adsorption of methylene blue onto jute fiber carbon: kinetics and equilibrium studies. *J. Colloid. Interf. Sci.* ***284***, 78 (2005)

[9] Kavitha, D., & Namasivayam, C. Experimental and kinetic studies on methylene blue adsorption by coir pith carbon. *Bioresource. Tecnol.* ***98***, 14 (2007)

[10] Li, Y., Du, Q., Liu, T., Peng, X., Wang, J., Sun, J., ... & Xia, L. Comparative study of methylene blue dye adsorption onto activated carbon, graphene oxide, and carbon nanotubes. *Chem. Eng. Res. Des.**91***, 361 (2013)
